# PredCMB: predicting changes in microbial metabolites based on the gene–metabolite network analysis of shotgun metagenome data

**DOI:** 10.1093/bioinformatics/btaf020

**Published:** 2025-01-15

**Authors:** Jungyong Ji, Sungwon Jung

**Affiliations:** Department of Health Sciences and Technology, GAIHST, Gachon University, Incheon 21999, Republic of Korea; Department of Genome Medicine and Science, Gachon University College of Medicine, Incheon 21565, Republic of Korea; Gachon Institute of Genome Medicine and Science, Gachon University Gil Medical Center, Incheon 21565, Republic of Korea

## Abstract

**Motivation:**

Microbiota-derived metabolites significantly impact host biology, prompting extensive research on metabolic shifts linked to the microbiota. Recent studies have explored both direct metabolite analyses and computational tools for inferring metabolic functions from microbial shotgun metagenome data. However, no existing tool specifically focuses on predicting changes in individual metabolite levels, as opposed to metabolic pathway activities, based on shotgun metagenome data. Understanding these changes is crucial for directly estimating the metabolic potential associated with microbial genomic content.

**Results:**

We introduce Predicting Changes in Microbial metaBolites (PredCMB), a novel method designed to predict alterations in individual metabolites between conditions using shotgun metagenome data and enzymatic gene–metabolite networks. PredCMB evaluates differential enzymatic gene abundance between conditions and estimates its influence on metabolite changes. To validate this approach, we applied it to two publicly available datasets comprising paired shotgun metagenomics and metabolomics data from inflammatory bowel disease cohorts and the cohort of gastrectomy for gastric cancer. Benchmark evaluations revealed that PredCMB outperformed a previous method by demonstrating higher correlations between predicted metabolite changes and experimentally measured changes. Notably, it identified metabolite classes exhibiting major alterations between conditions. By enabling the prediction of metabolite changes directly from shotgun metagenome data, PredCMB provides deeper insights into microbial metabolic dynamics than existing methods focused on pathway activity evaluation. Its potential applications include refining target metabolite selection in microbial metabolomic studies and assessing the contributions of microbial metabolites to disease pathogenesis.

**Availability and implementation:**

Freely available to non-commercial users at https://www.sysbiolab.org/predcmb.

## 1 Introduction

The microbiome, residing in various organs of the host body, plays a critical role in the host’s biological response to biochemical stimuli (Devaraj *et al.* 2013, Krautkramer *et al.* 2021). From this perspective, various studies have been carried out to investigate the interaction between microbiome and host biology including that of human. Recent studies have highlighted the significant influence of microbial metabolites on host health (Dayama *et al.* 2020). For instance, metabolic products of intestinal microorganisms have been linked to metabolic diseases such as obesity and diabetes, cardiovascular diseases, and neuropsychiatric disorders (Wijdeveld *et al.* 2020). Given their potential impact on host biology and pathogenicity, accurately identifying microbiome-derived metabolites is of great importance.

Conventional strategies for analyzing microbial metabolites include direct metabolomic profiling and analytical predictions based on metagenome sequencing data (Schirmer *et al.* 2019, Yachida *et al.* 2019). Techniques like mass spectrometry (MS) and liquid chromatography (LC) used for direct metabolomic profiling face limitations in metabolite coverage and high costs, as these technologies often only detect a limited range of metabolites. Analytical prediction tools, such as HUMAnN (Franzosa *et al.* 2018), infer the abundances of functional or metabolic pathways from microbial gene abundances. However, these methods typically estimate pathway abundances rather than metrics specific to metabolites.

Recently, new approaches were presented to predict disease phenotypes or microbial metabolic features based on training data, where predictive models of metabolites have been developed based on a paired dataset of metagenome and metabolome (Mallick *et al.* 2019, Le *et al.* 2020, Yin *et al.* 2020, Reiman *et al.* 2021). Despite their promise, these machine learning-based predictive methods are constrained by the limited availability of high-quality training datasets of paired microbiome and metabolome that comprehensively cover diverse biological and experimental conditions. The performance of such models heavily depends on the quantity and quality of training data, which is often restricted to specific conditions.

Another approach involves predicting microbial metabolite dynamics using genome-scale metabolic models (GEMs) of the microbiome, where flux-based metabolic dynamics can be assessed based on GEMs [see review articles (Ankrah *et al.* 2021, Heinken *et al.* 2021) on microbial GEM studies for more detailed information]. GEM-based analysis on specific microbial strains can be conducted with pre-built GEMs and constraint information for target biological contexts (Zelezniak *et al.* 2014, Kim *et al.* 2023), where pre-built GEMs can be publicly available in model repositories (King *et al.* 2016, Heinken *et al.* 2023). However, GEM-based analysis on microbial communities is not straightforward, as an integrated analysis of GEMs from microorganisms in the community needs to be conducted (Shoaie *et al.* 2015, Kumar *et al.* 2018) or a new pan-genome GEMs needs to be built from the metagenome data (Zorrilla *et al.* 2021, Guo *et al.* 2024). Both approaches demand substantial effort to build and optimize models for specific contexts.

Without such needs for target context-specific resources of training data or computational models like GEMs, utilizing known metabolic reaction information like the reporter metabolites (Patil and Nielsen 2005) method can be another choice of approach. However, this method has not been applied to metagenome data from microbial communities due to the lack of curated microbial metabolic reaction data suitable for such analyses.

In this study, we propose PredCMB, a novel method for quantitatively predicting changes in individual microbial metabolites between two conditions solely using shotgun metagenome sequencing data. PredCMB estimates metabolite changes based on the abundance changes of enzymatic gene families that are involved in the production and consumption of the metabolite as well as the concept of reaction abundance weight.

To show the validity of our proposed method, we used two publicly available paired datasets of shotgun metagenomics and metabolomics from inflammatory bowel diseases (IBDs) cohorts (Franzosa *et al.* 2019) and cohorts of gastrectomy for gastric cancer (Poyet *et al.* 2019). We evaluated the concordance between PredCMB’s predicted metabolite changes and experimentally measured changes. The comparative evaluation shows that our proposed method can predict correlated changes of metabolites with actual measurements, while concordantly predicting the metabolite classes that show major changes between given conditions.

The main contributions of this study are 2-fold: (i) the development of curated microbial metabolic reaction data suitable for network-based prediction methods, and (ii) the enhancement of network-based analysis with PredCMB, which accounts for both production and consumption reactions as well as reaction abundance weight, unlike previous methods that consider only production reactions. PredCMB provides a metagenome-based method for predicting metabolic changes, paving the way for novel microbiome research opportunities. It enables rapid identification of major metabolic shifts in the microbiome and offers directional insights for downstream metabolomic analyses.

## 2 Materials and methods

**Figure 1.**
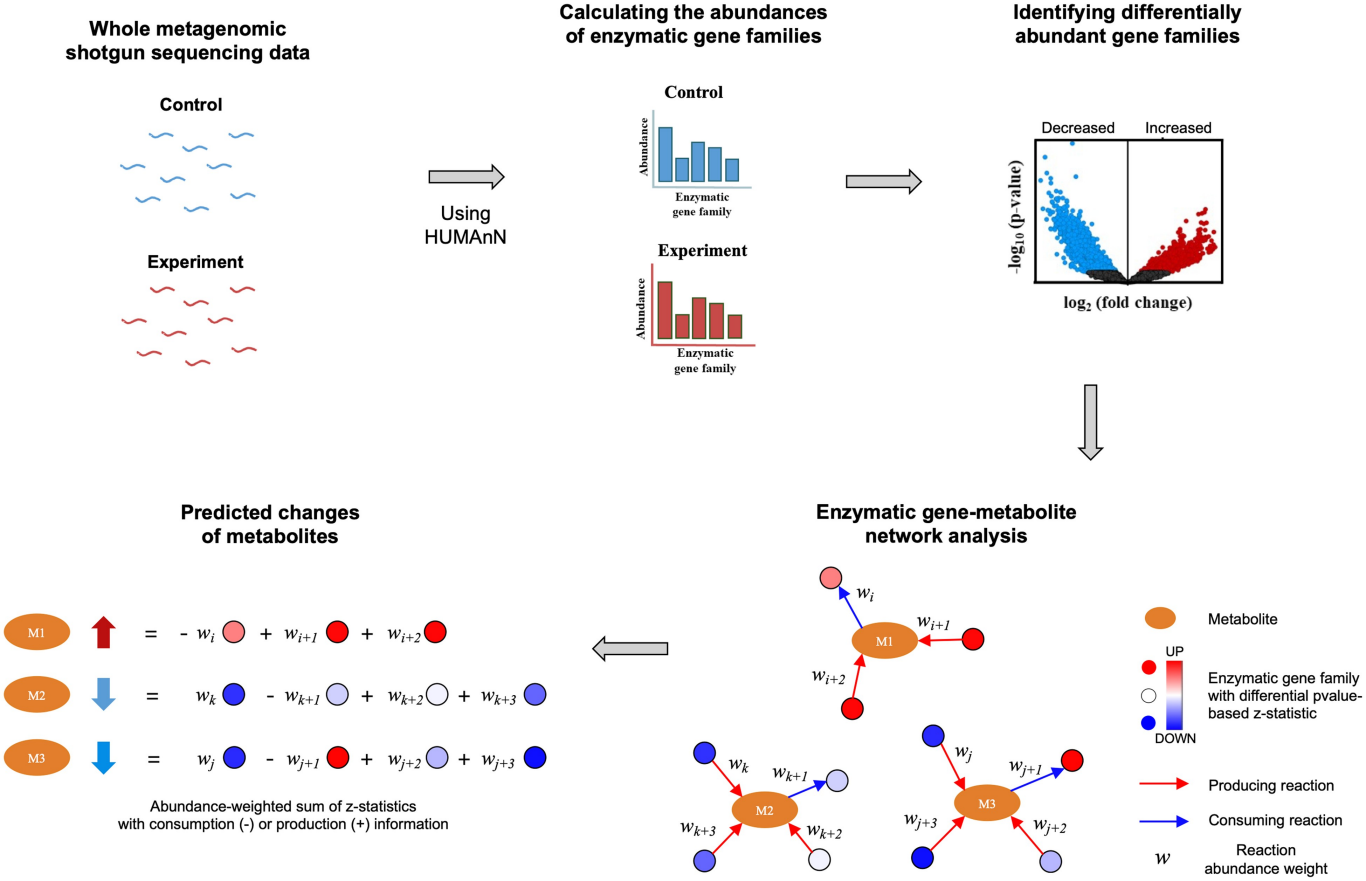
The conceptual workflow of the proposed method, PredCMB, illustrating the key steps involved in predicting changes in microbial metabolites based on shotgun metagenome data.

PredCMB uses shotgun metagenome sequencing data of two conditions (control and experiment) as input and gives predicted changes of metabolites through the enzymatic gene–metabolite network analysis based on differential abundances of enzymatic gene families between two conditions. The overall workflow of PredCMB is illustrated in Fig. 1.

First, the abundance of each known enzymatic gene family is profiled by mapping the shotgun metagenome sequencing data to the enzymatic gene families. Then, the differentiality of each enzymatic gene family is computed as a *P*-value by statistically comparing the abundances of the enzymatic gene family between the two conditions. Finally, the enzymatic gene-metabolic network analysis predicts the relative change in each metabolite between conditions, by summarizing the differentiality statistics based on *P*-values of enzymatic gene families contributing to the production and consumption of each metabolite, while incorporating the reaction abundance weight into the calculation.

### 2.1 Profiling the abundances of enzymatic gene families from microbiome

From shotgun metagenome sequencing data, the abundances of known enzymatic gene families are profiled by aligning sequencing reads to the UniRef90 (Suzek *et al.* 2015) protein family sequence database using the Biobakery3 (Beghini *et al.* 2021) pipeline, including Kneaddata, MetaPhlAn (Truong *et al.* 2017), and HUMAnN3 (Franzosa *et al.* 2018). UniRef90 defines protein families as groups of evolutionarily related protein-coding sequences that perform the same function, serving as a foundation for describing microbial gene family structures. Through the process of HUMAnN3 within Biobakery3, the gene family abundance is weighted based on the alignment quality and normalized by the length of both gene and alignment, then further normalized to copies per million (CoPM) units.

Since PredCMB focuses exclusively on enzymatic gene families, aligning sequence reads only to enzymatic genes can significantly reduce HUMAnN3’s computational runtime. To achieve this, we created a custom Enzyme Commission (EC)-filtered ChocoPhlAn database by filtering reference sequences in the UniRef90 database with assigned EC numbers. This custom EC-filtered database enabled a roughly 3-fold reduction in HUMAnN3’s runtime (data not shown). However, some performance degradation was observed in downstream metabolite change predictions (Supplementary Material Section SA). For this study, we opted to use the conventional HUMAnN3 pipeline with the full reference database to ensure optimal performance in the preparation of input data for subsequent analyses.

### 2.2 Identifying differentially abundant enzymatic gene families

The differentiality *P*-value of each enzymatic gene family can be computed by utilizing existing tools to evaluate differential expressions of next-generation sequencing data, and the PyDESeq2 (Muzellec *et al.* 2023) python package was used in this study to evaluate the differentiality based on the negative binomial model for sequence read-count information. Before evaluating differentiality *P*-values, the CoPM-normalized enzymatic gene family abundances are rounded to nearest integer values to mimic conventional integer-valued count data. Gene families that show zero abundances in >75% of samples are omitted from further analysis (Jonsson *et al.* 2016, Jonsson *et al.* 2017). The identification of differentially abundant enzymatic gene families (DAGs) can be performed with different thresholds of fold-changes in abundances and differentiality *P*-values. In our study, we declared DAGs as enzymatic gene families that show >2-fold changes in abundances (|log_2_(abundance ratio)| > 1) and the differentiality *P*-value < 0.05.

### 2.3 Construction of enzymatic gene–metabolite networks

The enzymatic gene–metabolite interaction data were compiled from the metabolic reactions cataloged in the Kyoto Encyclopedia of Genes and Genomes (KEGG) database (Kanehisa *et al.* 2017). Enzymes in KEGG reactions were mapped to their corresponding enzymatic gene families in UniRef90 (Suzek *et al.* 2015) using EC and KO numbers. For bidirectional metabolic reactions, the directionality was simplified by assigning each reaction a single direction for ease of analysis. The eQuilibrator tool (Flamholz *et al.* 2012) was used to determine the direction of bidirectional KEGG metabolic reactions. This determination was based on Gibbs free energy calculations, which leveraged the thermodynamic properties of biochemical compounds and reactions.

**Figure 2.**
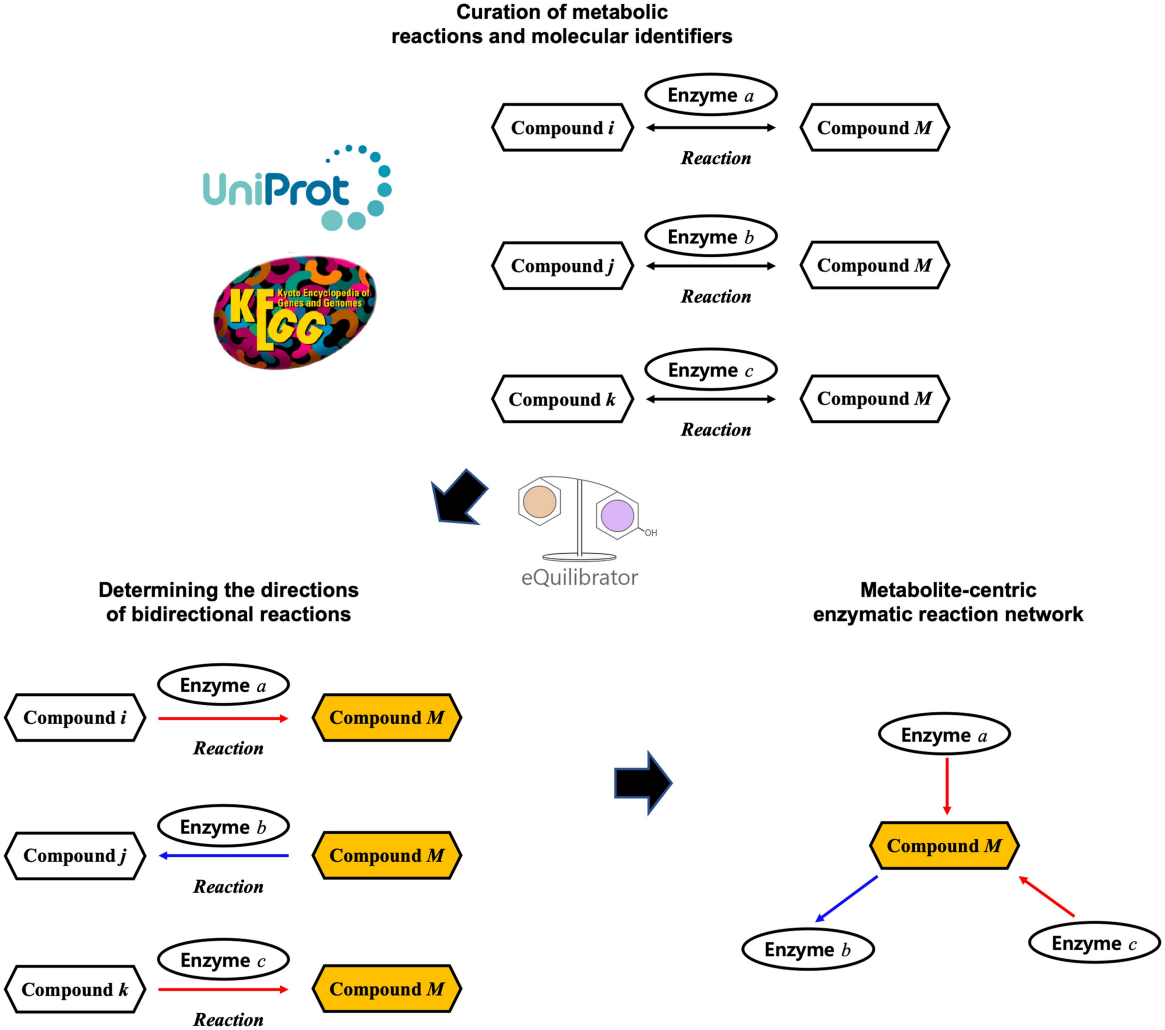
The process of constructing enzymatic gene–metabolite networks, where a metabolite-centric network component is built for each metabolite compound.

Enzymatic gene–metabolite networks were constructed from the compiled interactions through these processes, where a metabolite-centric network component is built for each metabolite compound by connecting enzymatic gene families that contribute to the production and consumption of the metabolite. The entire process of constructing enzymatic gene–metabolite networks is illustrated in Fig. 2. In total, 8298 metabolic reactions from KEGG were compiled, resulting in the construction of metabolite-centric network components for 3360 metabolite compounds.

### 2.4 Estimating metabolite changes and statistical significance using network information

To estimate the change of each metabolite abundance from the change of the microbial genome, the metabolite-centric network component is used along with the *P*-values of differentiality statistics of DAGs and their reaction abundance weights around the metabolite. In the metabolite-centric network, each metabolite has incoming interactions from enzymatic genes contributing to its production and outgoing interactions to enzymatic genes involved in its consumption. We assume that changes in metabolite abundance can be determined by summarizing the differentiality statistics of the relevant enzymatic genes, accounting for their roles in production or consumption. In this study, the differentiality statistic of each enzymatic gene is represented by its *P*-value.

To quantify these changes, the *P*-values of enzymatic gene families are converted to Z-scores, which reflect the magnitude and direction of abundance changes relative to controls. In this process of *P*-value conversion to Z-scores, we convert the *P*-values of increased gene families to positive Z-scores and the *P*-values of decreased gene families to negative Z-scores. Specifically, the *P*-value *P_i_* of the *i*th gene family is modified as follows:

- *P_i_*/2 if the abundance of the gene family is increased.
- *P_i_*/2 if the abundance of the gene family is decreased.

This makes increased gene families have modified *P*-values with the range between 0 and 0.5, and decreased gene families have modified *P*-values with the range between 0.5 and 1. The modified *P*-values are then converted to Z-scores that matches the modified *P*-values from the inverse normal cumulative distribution, eventually making positive Z-scores for increased gene families and negative Z-scores for decreased gene families. Based on the Z-scores of enzymatic gene families, the Z-score Z_metabolite_ that represents the relative change of a metabolite is calculated as follows:


(1) $$Z_{\mathrm{metabolite}}=\frac{1}{k}\sum_{i=1}^{k}R(i)\cdot w_{i}\cdot Z_{i},$$

where *k* is the number of enzymatic gene families associated with the metabolite, *R*(*i*) is a function that returns 1 for an enzymatic gene families producing the metabolite and −1 for those consuming it. *w_i_* is a reaction abundance weight for the enzymatic gene family *i*, calculated as the relative abundance of the gene family in control samples as follows:


(2) $$w_{i}=\;\frac{{\text{abundance}}_{i}^{\text{avr}}}{\sum_{j=1}^{E}{\text{abundance}}_{j}^{\text{avr}}},$$

where *E* is the total number of enzymatic gene families and ${\text{abundance}}_{i}^{\text{avr}}$ represents the average abundance of an enzymatic gene family *i* in control samples. This weight reflects the relative contribution of a gene family’s reaction under the baseline (control) condition.

To evaluate the statistical significance of Z_metabolite_, we use the normal distribution to obtain the corresponding *P*-value. However, the distribution of Z_metabolite_ may not match the exact normal distribution. Thus, Z_metabolite_ values are adjusted first based on the null distribution of Z_metabolite_ values that is obtained by randomly building 1000 metabolite-centric network components with randomly selected *k* enzymatic gene families as follows while preserving the original count of producing and consuming gene families:

(3) $$Z_{\mathrm{NullAdj}}=\frac{\left(Z_{\mathrm{metabolite}}-\mu_{k}\right)}{\sigma_{k}},$$

where $\mu_{k}$ and $\sigma_{k}$ indicate the mean and standard deviation of the null distribution Z-scores from random building of metabolite-centric network components. Finally, the statistical significance *P*-value for Z_metabolite_ is determined by the right-tail *P*-value that corresponds to Z_NullAdj_ from the standard normal distribution if it is positive, and by the left-tail *P*-value if Z_NullAdj_ is negative.

### 2.5 Estimating metabolite changes and statistical significance using network information

To identify metabolite classes with significant increase or decrease, we performed enrichment analysis based on the Wilcoxon rank sum test by ranking Z_metabolite_ values of individual metabolites. A metabolite class was considered significantly increased or decreased if its metabolites consistently ranked particularly high or low in the rank-based comparison between metabolites within the class and those outside the class. Two-tailed nominal *P*-values were evaluated and corrected using the Benjamini-Hochberg (BH) method, and the false discovery rate (FDR) of 0.05 was used as significance threshold in this study. We used the categorization of metabolite classes based on the chemical classes level information from Human Metabolome Database (HMDB) (Wishart *et al.* 2022), and metabolite classes with <5 metabolites were excluded in this study.

## 3 Results

### 3.1 Configuration for evaluating PredCMB with benchmark datasets

For the benchmark evaluation of the proposed method PredCMB, we used two publicly available paired datasets of shotgun metagenome and metabolome from the PRISM IBD cohort study (Franzosa *et al.* 2019) and the cohort of gastrectomy for gastric cancer (Poyet *et al.* 2019). The IBD cohort dataset was generated from the stool samples of the 68 patients with Crohn’s disease (CD), 53 patients with Ulcerative colitis (UC), and 34 subjects of non-IBD controls. The dataset of Gastrectomy cohort was generated from the stool samples of the 42 subjects with the history of gastrectomy for gastric cancer and 54 healthy control subjects. The metabolomics data for the IBD cohort were derived from untargeted metabolomics, encompassing 8848 metabolic features. However, only a subset of these features was annotated with known metabolites or metabolite classes: 3829 features were annotated with 474 metabolite classes, and 466 features were annotated with 386 metabolites. The Gastrectomy metabolome data are based on targeted metabolomics, where 524 metabolic features are included. All 524 metabolic features are annotated with known 524 metabolite compounds as they are targeted, and 384 of them are annotated with 76 metabolite classes. In the subsequent benchmark analysis, we compared the predicted changes in metabolites and metabolite classes by PredCMB with the experimentally measured changes. These comparisons were made for three contrasts: CD versus controls, UC versus controls, and gastrectomy versus controls. For comparative analysis, the reporter metabolite method was also used to predict metabolic changes for these benchmarks. The analysis using the reporter metabolite method was conducted using the Piano Bioconductor R package (Patil and Nielsen 2005, Varemo *et al.* 2013), which was built by the developers of the reporter metabolite method.

### 3.2 Differentially abundant microbial enzymatic gene families in benchmark data

We evaluated the abundances of enzymatic gene families in all the benchmark metagenome samples using HUMAnN3. From evaluating the differential abundances of the enzymatic gene families between CD versus control, the abundances of 18 549 enzymatic gene families were identified to be significantly increased and 58 656 enzymatic gene families showed significantly decreased abundances. Between UC and control, the abundances of 5312 enzymatic gene families were increased while 45 729 enzymatic gene families showed decreased abundances (Supplementary Table S1). Out of all the identified DAGs, the majority (76% in CD, 90% in UC) of DAGs showed significantly decreased abundances in IBD-subtypes relative to control. This can be relevant with the result of the reference study (Franzosa *et al.* 2019) where the majority (71%) of significantly changed metabolites in IBD cases showed decreased abundances compared to controls. This can imply that the changes in stool metabolites have certain level of consistency with the changes in relevant microbial enzymatic genes. The number of DAGs from the Gastrectomy cohort was relatively smaller than that of the IBD cohort (4513 increased DAGs and 3217 decreased DAGs), and the numbers of increased DAGs and decreased DAGs did not show such huge difference from the IBD cohort. Evaluation of metabolite class changes in the Gastrectomy cohort revealed no statistically significant increases or decreases in any metabolite class (Supplementary Fig. S1), although some classes demonstrated a modest decrease. The smaller number of DAGs in the gastrectomy cohort may explain the mild changes observed in metabolite abundances.

### 3.3 Concordance between predicted and measured metabolite changes

**Figure 3.**
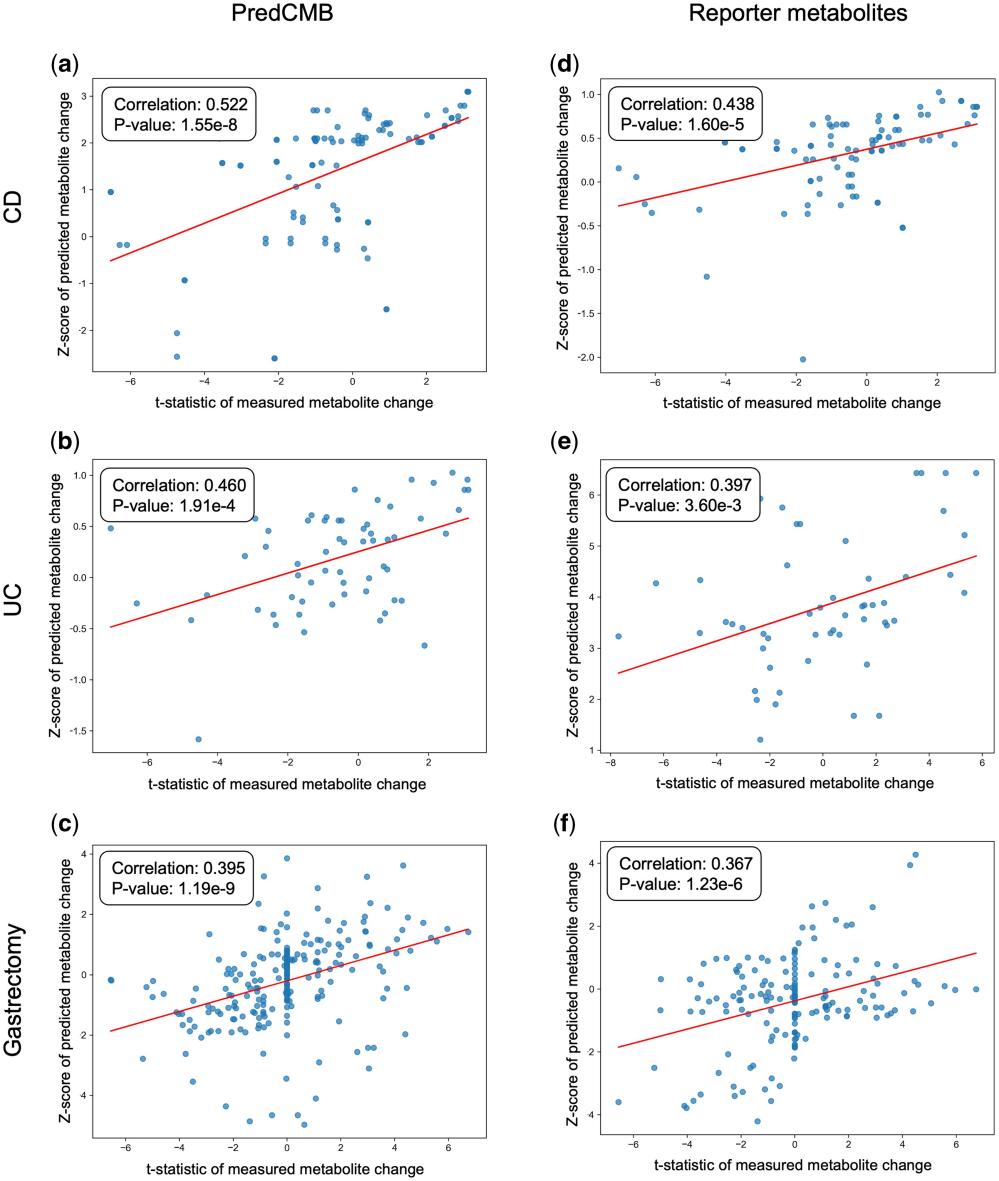
Correlation between metabolite change statistics from the actual measurements and predictions using PredCMB (a–c) and the reporter metabolites method (d–f). The *t*-statistics of measured changes from the Gastrectomy data (c, f) include many zero values (no change in metabolite abundance), as the data includes metabolites with zero abundance from its targeted metabolomics.

We compared the Z-scores of predicted metabolite changes generated by PredCMB with the t-statistics of metabolite changes derived from actual measurements (Fig. 3a–c). For each benchmark cohort data, only the commonly identified metabolites were compared, where 103, 61, and 221 metabolites were common for CD, UC, and Gastrectomy cohorts accordingly. The predicted metabolite changes using PredCMB show correlation coefficients from 0.395 to 0.522 from these three comparisons, where all correlations satisfy statistical significance *P*-value < 0.05.

We also compared the predicted changes of metabolites using the reporter metabolites method with their measured changes, where 90, 52, and 165 metabolites were common and compared for CD, UC, and Gastrectomy cohorts accordingly (Fig. 3d–f). The reporter metabolite method also shows meaningful correlations from 0.367 to 0.438 across benchmarks, where they also satisfy statistical significance with low *P*-values. When comparing the results of PredCMB and the reporter metabolites method, it is notable that PredCMB consistently achieved higher correlation coefficients across all benchmark datasets.

### 3.4 Concordance between predicted and measured changes in metabolite classes

The predicted changes in metabolite classes using PredCMB and the reporter metabolites method were compared with the actual measurements to evaluate their concordance. Only the metabolite classes common across predictions and measurements were analyzed.

**Figure 4.**
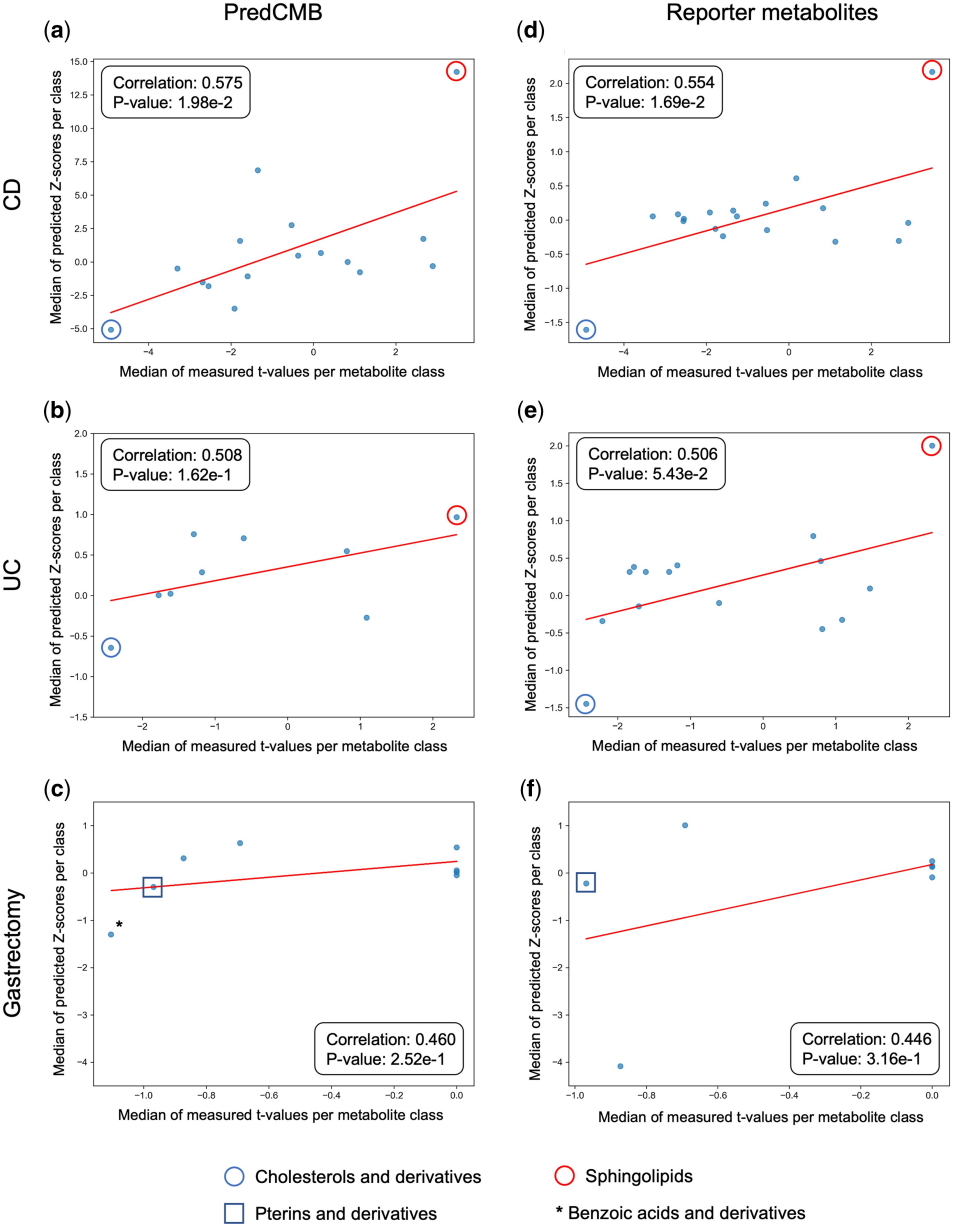
Correlation of metabolite class change statistics between actual measurements and predictions using PredCMB (a–c) and the reporter metabolites method (d–f). Each data point represents a metabolite class, with its value corresponding to the median change statistic of its member metabolites.

Figure 4 illustrates scatter plots of the median change statistics of metabolite classes (based on their member metabolites) relative to controls, comparing predictions with actual measurements. Predicted changes and the changes from the actual measurements show positive correlations for both methods across all benchmark datasets. All predictions show certain level of correlations from 0.446 to 0.575, while statistically significant *P*-values (<0.05) were observed only from the CD cohort of the IBD benchmark data. This can be partly due to the small number of comparable metabolite classes between predictions and the actual measurements. It is notable that PredCMB showed higher correlations than the reporter metabolites method across all benchmark datasets. The results also highlighted the concordance in metabolite classes with substantial changes. The class of sphingolipids showed the largest increase, while the class of cholesterol and its derivatives showed the largest decrease among the commonly identified metabolite classes in both predictions and actual measurements (Fig. 4a, b and d–e). For the Gastrectomy cohort, the class of pterins and derivatives exhibited the largest decrease in actual measurements among the common metabolite classes. PredCMB successfully identified this class as the most decreased, unlike the reporter metabolites method (Fig. 4c and f). In addition, the class of benzoic acids and derivatives, identified only by PredCMB, showed a median change statistic lower than that of pterins and derivatives, consistent with the actual measurement (Supplementary Fig. S1).

**Figure 5.**
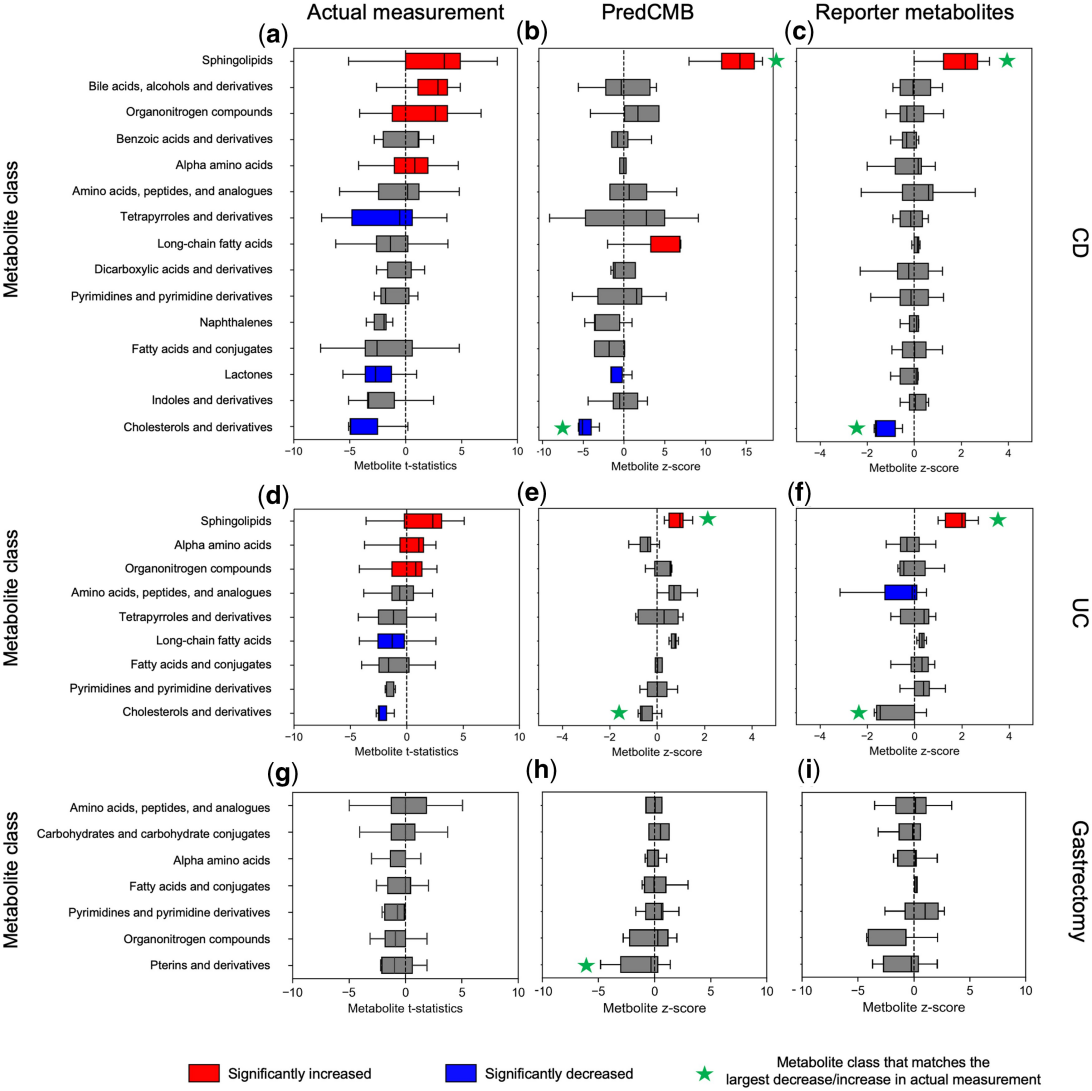
Change statistics of metabolite classes from predictions and actual measurements. Each boxplot represents the change statistics of a metabolite class based on its individual metabolites, with the box indicating 25th and 75th percentiles and the center line denoting the median. The increase or decrease of each metabolite class was determined based on the median value of the change statistics of its member metabolites. Metabolite classes with statistically significant changes are highlighted in red (increased) or blue (decreased). For each benchmark, only metabolite classes common to actual measurements and all predictions are shown. (a–c) CD of the IBD cohort with 15 metabolite classes. (d–f) UC of the IBD cohort with nine metabolite classes. (g–i) Gastrectomy cohort with seven metabolite classes.

Figure 5 illustrates the boxplots for the change statistics of metabolite classes based on their individual metabolites. For the CD benchmark, sphingolipids as well as cholesterols and derivatives showed the largest increase and decrease (based on median values) among the common metabolite classes from both predictions and the actual measurement, while satisfying statistical significance for their changes. For the UC group, sphingolipids showed the largest increase with statistical significance from both predictions and measurement. Cholesterols and derivatives showed a statistically significant decrease only from the actual measurement, but it showed the largest decrease from both predictions and measurement among these metabolite classes. For the Gastrectomy cohort data, no metabolite class showed statistically significant changes in the actual measurements or predictions. However, PredCMB identified pterins and derivatives as the most decreased class among these common classes, consistent with the actual measurements.

There can be inherent discrepancies between the change statistics of measured metabolites and our metagenome-based change predictions due to environmental factors. The measured metabolites from subjects’ stool samples can contain variations from subjects’ heterogeneity such as different diets, lifestyles, and health conditions. However, the metagenome-based prediction estimates the metabolic changes that are caused only by the constitution of microbiome, based on their DNA contents. In addition, the coverage of metabolites from experimental measurements and computational predictions based on curated metabolic reactions can be largely different as mentioned in the previous section where relatively small fractions of metabolites are comparable for validation. Despite these challenges, our results demonstrate that metagenome-based predictions of metabolite changes using PredCMB are correlated with actual measurements. PredCMB showed especially strong concordance for metabolite classes with substantial changes. Moreover, additional considerations of metabolite-consuming reactions and reaction abundance weights in PredCMB enabled predictions with further improved correlations with actual measurements than the previous method that considers only the metabolite-producing reactions. Thus, our proposed method can be a beneficial tool in predicting the changes in microbial metabolites, especially when the environment consists of complex microbial communities and the change in microbial contents is a major factor that causes metabolic changes.

## 4 Discussion

We introduce a novel method, PredCMB, which predicts changes in individual microbial metabolites based on shotgun metagenome data from two different conditions. PredCMB utilizes networks of microbial enzymatic genes and their interactions with metabolites to infer these changes. To evaluate its predictive capabilities, we used publicly available paired datasets of shotgun metagenomics and metabolomics from IBD studies and a gastrectomy cohort as benchmarks. We compared the predicted changes of metabolites with the changes from actual measurements based on the benchmarks. Comparisons of predicted metabolite changes with measured changes demonstrated positive and statistically significant correlations, with PredCMB achieving higher correlations than a previous method. In addition, predicted changes in metabolite classes, derived by summarizing individual metabolite predictions, were compared with measured changes. From the comparison, the predicted changes of metabolite classes showed positive correlations with their measured changes. Notably, metabolite classes exhibiting the largest increases or decreases in predictions were largely consistent with those from measurements. PredCMB was able to identify an additional metabolite class with further decreased metabolites, underscoring its improved performance. These findings suggest that PredCMB provides concordant predictions of metabolic changes with actual measurements, offering improved concordance over previous methods.

Using PredCMB can provide us meaningful opportunities in microbial studies by enabling the prediction of metabolic changes solely based on microbial genomic contents. For example, we can adjust the coverage of metabolomics measurements based on the prediction using PredCMB for more effective experiments with lower costs. Another benefit of PredCMB is that it works solely based on shotgun metagenome data, and it does not require reference metabolomics measurements or computational models of target biological contexts as other approaches based on machine learning predictions or GEM approaches do. A limitation of our study is that PredCMB relies on the statistical analysis of differentially abundant enzymatic gene families, which require multiple samples per group for statistical testing. As such, it cannot provide predictions for individual samples. A more inherent limitation is that prediction of metabolic changes based on metagenome information cannot reflect other contributing factors in stool samples than microbiome. In addition, the limited coverage of common metabolites between predictions and measurements may have constrained the completeness of our evaluations. Future studies will address this issue by incorporating improved metabolomics technologies and additional benchmark datasets as they become available. Many recent studies reveal the effects of microbiome activities in host pathogenesis and phenotypic changes, and we believe that our method can provide certain advances in studying the biological mechanisms of microbial metabolites.

## Supplementary Material

btaf020_Supplementary_Data

## Data Availability

No new data were generated or analyzed in support of this research.
