# Supplementary material for: PredCMB: predicting changes in microbial metabolites based on the gene–metabolite network analysis of shotgun metagenome data
Source: Bioinformatics. 2025 Jan 15;41(1):btaf020. doi: 10.1093/bioinformatics/btaf020 (PMC11771765; doi:10.1093/bioinformatics/btaf020)
Supplement: btaf020_Supplementary_Data [file btaf020_supplementary_data.zip › dfd87_SupplementaryDocument.pdf]

*Supplementary material for:*

# PredCMB: Predicting changes in microbial metabolites based on the gene-metabolite network analysis of shotgun metagenome data

## A. Using HUMAnN3 with a custom EC-filtered ChocoPhlAn Database

To prepare input for PredCMB, HUMAnN3 is used to profile the abundance of enzymatic gene families from raw FASTQ files. While conventional HUMAnN3 workflows profile all gene families, PredCMB requires abundance information only for enzymatic gene families.

In order to run HUMAnN3 with limited gene family coverage to enzymatic gene families, a custom ChocoPhlAn DB with only enzymatic gene family sequences is necessary for the procedure of nucleotide sequence alignment, and Enzyme Commission (EC)-filtered UniRef DB should be used for the process of amino acid sequence alignment. EC-filtered UniRef 90/50 databases are provided in HUMAnN3, while no custom ChocoPhlAn DB with only enzymatic gene families is provided. HUMAnN3 can be used only with the EC-filtered UniRef databases, but using a custom EC-filtered ChocoPhlAn DB can further reduce the running time of HUMAnN3.

We created a custom EC-filtered ChocoPhlAn DB from the original ChocoPhlAn DB (downloaded April 12, 2024) by filtering reference sequences based on EC numbers included in the EC-filtered UniRef90 DB. A corresponding annotation file was also generated. (Download of the files and usage instructions are available at PredCMB webpage: https://www.sysbiolab.org/predcmb)

Using the custom EC-filtered ChocoPhlAn DB reduced HUMAnN3 runtime to approximately one-third of the original runtime, though this improvement depends on input data and the computing environment.

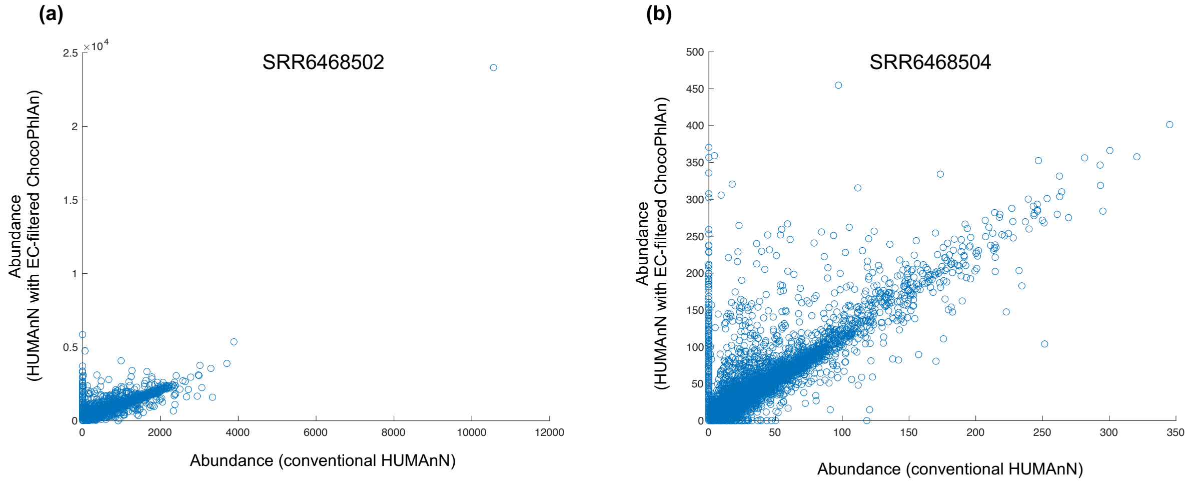


Supplementary Figure A1. Scatter plots comparing estimated enzymatic gene abundances from HUMAnN3 with and without using the custom EC-filtered ChocoPhlAn DB. (A) Results for sample SRR6468502 from the IBD cohort data. (B) Results for sample SRR6468504 from the IBD cohort data

While using the custom EC-filtered ChocoPhlAn DB significantly reduced runtime, discrepancies were observed in the estimated abundances of enzymatic gene families compared to those obtained with the conventional HUMAnN3 pipeline. For example: For sample SRR6468502, the correlation coefficient between abundances estimated using the custom DB and the conventional DB was 0.8915 (Supplementary Figure A1(a)), while the correlation coefficient was 0.2072 for the sample SRR6468504 (Supplementary Figure A1(b)). From Supplementary Figure A1, most enzymatic gene families exhibited high correlations between the two approaches. However, some gene families were overrepresented in the custom DB results, particularly those with zero abundance

from the case of the conventional DB but non-zero abundance when the custom DB was used. This suggests that some sequence reads may be false-positively misaligned to enzymatic gene families due to the absence of correct reference sequences in the custom EC-filtered DB.

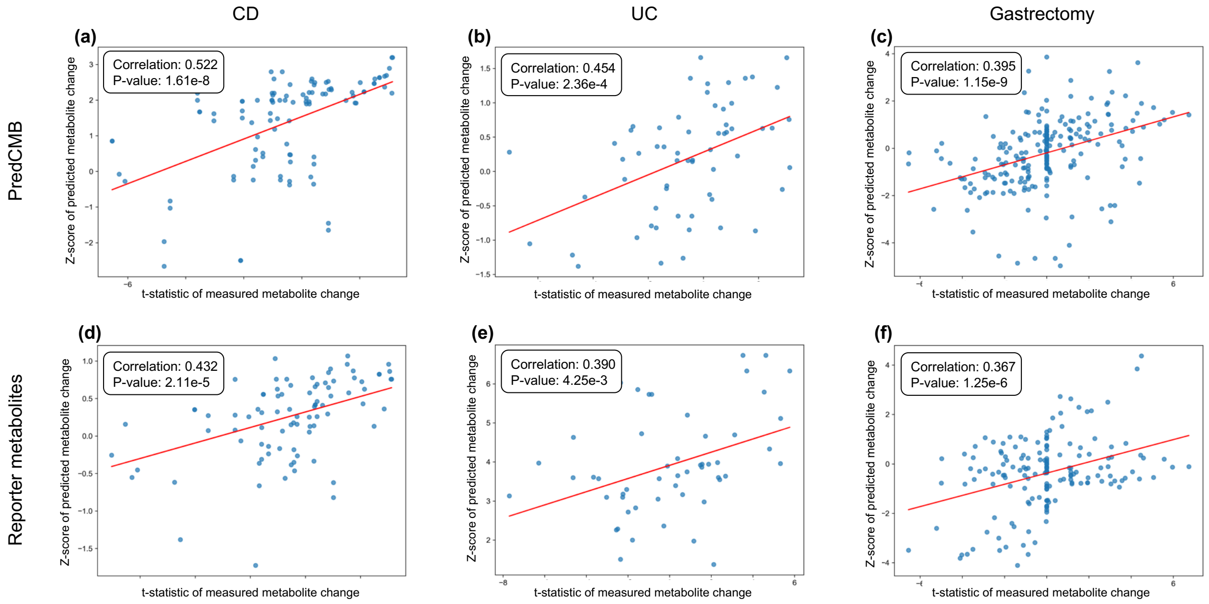


Supplementary Figure A2. Correlation between metabolite change statistics from the actual measurements and predictions using PredCMB (a – c) and the reporter metabolites method (d – f).

Supplementary Table A1. Correlations between metabolite change statistics from the actual measurements and predictions.

| | CD | | UC | | Gastrectomy | |
|---|---|---|---|---|---|---|
| | Conventional HUMAnN3 | Using EC-filtered ChocoPhlAn | Conventional HUMAnN3 | Using EC-filtered ChocoPhlAn | Conventional HUMAnN3 | Using EC-filtered ChocoPhlAn |
| PredCMB | 0.522 | 0.522 | 0.460 | 0.454 | 0.395 | 0.395 |
| Reporter metabolites | 0.438 | 0.432 | 0.397 | 0.390 | 0.367 | 0.367 |

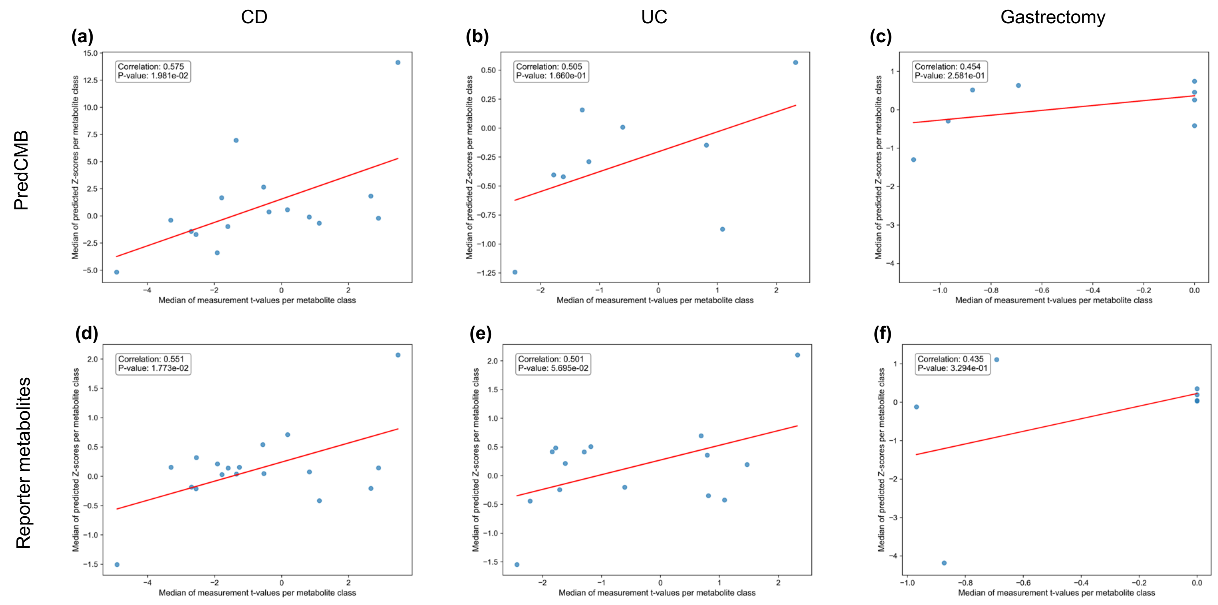


Supplementary Figure A3. Correlation between metabolite class change statistics from actual measurements and predictions using PredCMB (a – c) and the reporter metabolites method (d – f). Each data point represents a metabolite class, with its value corresponding to the median change statistics of its member metabolites.

Supplementary Table A2. Correlations between metabolite class change statistics from actual measurements and predictions.

| | CD | | UC | | Gastrectomy | |
|---|---|---|---|---|---|---|
| | Conventional HUMAnN3 | Using EC-filtered ChocoPhlAn | Conventional HUMAnN3 | Using EC-filtered ChocoPhlAn | Conventional HUMAnN3 | Using EC-filtered ChocoPhlAn |
| PredCMB | 0.575 | 0.575 | 0.508 | 0.505 | 0.460 | 0.454 |
| Reporter metabolites | 0.554 | 0.551 | 0.506 | 0.501 | 0.446 | 0.435 |

Using the custom EC-filtered ChocoPhlAn DB during input preparation led to decreased correlations between predicted and measured metabolite changes compared to using the conventional HUMAnN3 pipeline (Supplementary Figure A2, Supplementary Table A1). Similar decreases were observed for metabolite class changes (Supplementary Figure A3, Supplementary Table A2).

Although the performance reduction observed with the custom EC-filtered ChocoPhlAn DB was not substantial, it highlights the potential trade-off between runtime efficiency and analytical accuracy. Variability in results may depend on the dataset, and caution is advised when using the custom EC-filtered ChocoPhlAn DB for HUMAnN3, particularly if high precision in downstream analyses is required.

Supplementary Table 1. The number of identified DAGs from the benchmark data sets

| Comparison | Increased DAGs | Decreased DAGs |
|---|---|---|
| CD VS. Control | 18,549 | 58,656 |
| UC VS. Control | 5,312 | 45,729 |
| Gastrectomy VS. Control | 4,513 | 3,217 |

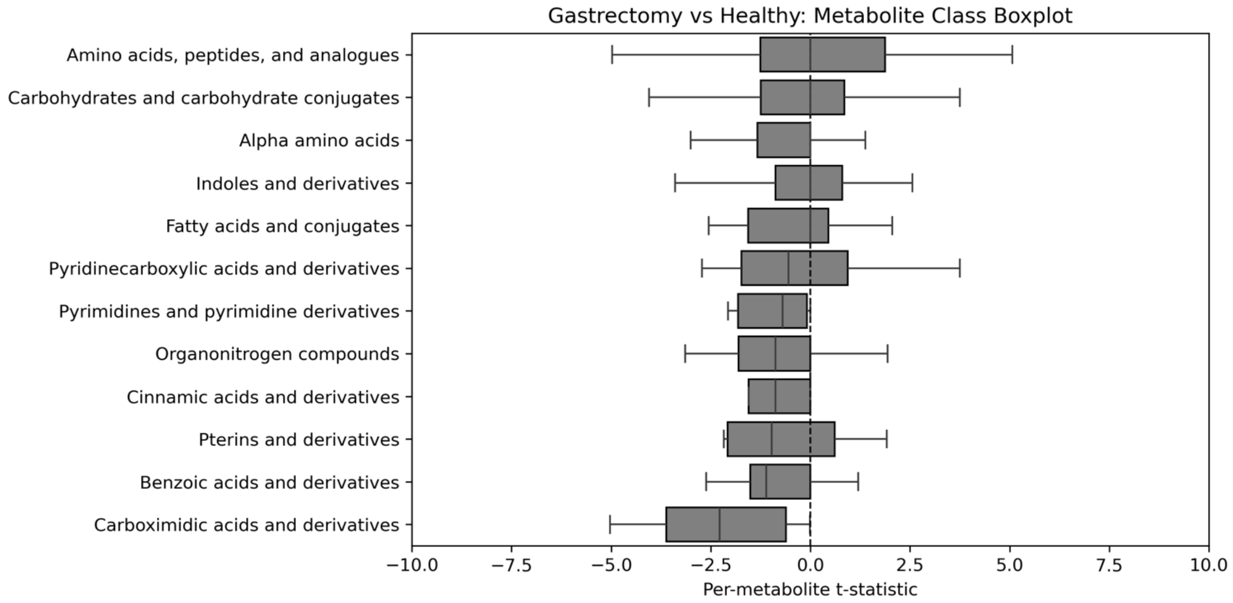


Supplementary Figure 1. Change statistics (t-statistics) of metabolites for each metabolite class from the Gastrectomy cohort compared to controls. Each boxplot represents the change statistics of a metabolite class based on its individual metabolites, with the box indicating the 25th and 75th percentiles and the center line denoting the median. No metabolite class exhibited a statistically significant change based on the Wilcoxon rank-sum test.
